# A multi‐isotope investigation of diet and subsistence amongst island and mainland populations from early medieval western Britain

**DOI:** 10.1002/ajpa.23127

**Published:** 2016-11-17

**Authors:** Katie A. Hemer, Angela L. Lamb, Carolyn A. Chenery, Jane A. Evans

**Affiliations:** ^1^ Department of ArchaeologyUniversity of SheffieldS1 4ETUnited Kingdom; ^2^ NERC Isotope Geosciences LaboratoryBritish Geological SurveyKeyworthNottinghamNG12 5GGUnited Kingdom

**Keywords:** Isle of Man, palaeodietary reconstruction, post‐Roman period, sulphur, Wales

## Abstract

**Objectives:**

This is the first investigation of dietary practices amongst multiple early medieval populations (AD 500–1000) from Wales and the Isle of Man using carbon, nitrogen, and sulphur isotope analysis. The analysis will illuminate similarities or differences between the diets and subsistence strategies of populations occupying different geographical regions, specifically those living in marginal coastal regions in comparison to inland populations well‐connected to ecclesiastical centres and high‐status settlements.

**Materials and Methods:**

One hundred and two human skeletons were sampled for carbon and nitrogen isotope analysis, and 69 human skeletons were sampled for sulphur isotope analysis from nine cemetery sites from western Britain (Isle of Man = 3, southwest Wales = 4, southeast Wales = 2). Thirteen faunal skeletons from St Patrick's Chapel (southwest Wales) were sampled for carbon, nitrogen, and sulphur isotope analysis.

**Results:**

Human δ^13^C values range from −19.4‰ to −21.2‰ (δ^13^C mean=−20.4 ±0.4‰, 1σ, n = 86), and δ^15^N values range from 9.1‰ to 13.8‰ (δ^15^N mean = 10.8 ± 0.9‰, 1σ, *n* = 86). δ^34^S values range from 1.2‰ to 18.4‰ (δ^34^S mean = 11.6 ± 4.5‰, 1σ, *n* = 66). Significant differences were noted between the mean δ^13^C, δ^15^N and δ^34^S values according to geographic region: Isle of Man (δ^13^C = −20.7 ± 0.4‰, δ^15^N = 11.4 ±0.6‰, *n* = 13/86; δ^34^S mean = 17.1 ±0.6, *n* = 4/66), southwest Wales (δ^13^C = −20.5 ± 0.4‰, δ^15^N = 11.0 ±1‰, *n* = 32/86; δ^34^S = 16.1 ± 2.1, *n* = 21/66), and southeast Wales (δ^13^C =−20.3 ±0.4‰, δ^15^N = 10.4 ±0.7‰, *n* = 41/86; δ^34^S= 8.8 ±3‰, *n* = 41/66). Faunal δ^13^C values range from −23.1‰ to −21.2‰ (δ^13^C mean= −22.1 ±0.5‰, 1σ, *n* = 13), and δ^15^N values range from 6.3‰ to 9.8‰ (δ^15^N mean = 7.3 ± 1.1‰, 1σ, *n* = 13). δ^34^S values range from 4.7‰ to 18.4‰ (δ^34^S mean= 16.3 ± 3.6‰, 1σ, *n* = 13).

**Conclusions:**

The data reveal a reliance on terrestrial protein, however differences are observed between the resource consumption of populations from southwest Wales and the Isle of Man in comparison to the populations from southeast Wales. Populations from the west coast have a marine sulphur signature that reflects their coastal proximity and may also include a reliance on seaweed as a fertiliser/food source. Populations in the southeast were connected to ecclesiastical centres and high‐status settlements and had access to inland‐grown produce. The data add support to the suggestion that δ^34^S can be used as a mobility indicator.

## 1 Introduction

The reconstruction of diet amongst early medieval (5th–11th century AD) populations from Britain has, to date, focused on Anglo‐Saxon populations from England. One of the earliest studies undertaken over a decade ago applied carbon and nitrogen isotope analysis to the early Anglo‐Saxon cemetery population of Berinsfield, Oxfordshire, and sought to investigate a correlation between diet and status (Privat & O'Connell, 2001). Another study saw the analysis of the late‐4th to mid‐6th century AD population from Queenford Farm, Oxfordshire and revealed gendered dietary differences relating to roles within society (Fuller, Fuller, Harris, & Hedges, 2006). Other studies of Anglo‐Saxon populations include the analysis of the 4th–7th century AD cemetery of Wasperton on the River Avon (Montgomery, Evans, Chenery, & Müldner, 2009), and the middle Anglo‐Saxon burial ground of Belle Vue House, York (Müldner & Richards, 2007). Mays and Beavan (2012) collated carbon and nitrogen isotope data for 76 individuals from 18 early Anglo‐Saxon cemeteries from England to investigate whether access to dietary resources was determined by the geographical location of the populations (i.e., inland, coastal, riverine), or the age or sex of the individuals. Pictish/Medieval (6th–15th century AD) populations from Portmahomack, Scotland have also recently been analysed (Curtis‐Summers, Montgomery, & Carver, 2014). All of the aforementioned studies relied on the application of carbon and nitrogen isotope analysis only. Sulphur isotope analysis is, however, increasingly being applied to such studies and can offer further insight into palaeodietary reconstruction and, potentially, to mobility studies (Richards, Fuller, & Hedges, 2001; Nehlich et al., 2011, p. 4964).

In contrast to the palaeodietary analysis of populations from Anglo‐Saxon England, only one investigation of diet amongst early medieval populations from Wales has been undertaken to date, primarily because of poor skeletal preservation amongst populations from western Britain. Five adult individuals excavated from the 10th‐century monastic site of Ty Newydd, on Bardsey Island in northwest Wales were subject to carbon and nitrogen isotope analysis, and the results revealed a diet reliant on terrestrial resources (Arnold, 1998). Given the dearth of comparative studies for western Britain, there is a considerable lacuna in our understanding of dietary practices amongst early medieval populations from Wales and the Isle of Man that could be addressed through stable isotope analysis. There is all the more need for a palaeodietary isotope investigation of these populations since archaeological evidence for subsistence practices is also limited due to a scant amount of settlement evidence from this region (Edwards, Lane, & Redknap, 2011). This study will therefore investigate the diets and resource consumption of populations from Wales and the Isle of Man through the application of carbon, nitrogen and sulphur isotope analysis to nine cemetery populations from three specific geographical regions; the Isle of Man, southwest Wales (Pembrokeshire), and southeast Wales. Consideration will be given to whether populations in these different regions relied on the same dietary resources, or whether location may have influenced access to, and consumption of, different foodstuffs and the reasons for this. The isotope data will be interpreted in the context of evidence from the historical and archaeological record to better understand such an essential part of everyday life of those communities living in early medieval western Britain.

### 1.1 Diet and subsistence in early medieval western Britain

The British cleric, Gildas, writing in the mid‐6th century AD provides a contemporaneous account of life in early medieval Britain in his *De Excidio Britanniae* (Winterbottom, 1978). According to Gildas, the island of Britain occupied “the end of the world” and had wide plains and hills “excellent for vigorous agriculture” (Winterbottom, 1978, p. 17). Mountains were used for “alternate” pasturage implying that transhumance—whereby farmers and livestock would spend their time in the hills on a summer farm, and would return in winter to a lowland farm—was practiced in the 6th century (Davies, 1982, p. 40; Roberts, 1959). We also hear from Gildas of constant flowing fountains, “brilliant rivers” and lakes flowing “with a cold rush of living water” (Winterbottom, 1978, p. 17). The people of early medieval western Britain were therefore supported by a rich and diverse landscape, which allowed them to grow crops and support livestock including sheep, pigs, goats, and cattle. The number of references to cattle in written sources suggests that cattle were central to the agrarian economy and were more important than other animals, and we hear of the severe consequences of cattle disease and cattle raiding (Davies, 1982, p. 39). Indeed, exchanges of land and compensation were valued, and payments made, in terms of cattle (Davies, 1978, p. 53). The importance of cattle is further reflected by the number of references to milk and dairy produce; the late‐10th century Latin learning aid *De Raris Fabulis*, written either in Wales or Cornwall, lists a range of vernacular terms for milk products suggesting a variety of diary produce and widespread use (Charles‐Edwards, 2013, p. 647; Davies, 1982, p. 35). Writing in the late‐12th century, the cleric Giraldus Cambrensis in his “Journey through Wales” notes that the people of Wales enjoyed a plentiful supply of meat and poultry, but consumed only a single meal a day that was served on a large, rolled‐out piece of thin baked bread akin to a modern‐day pizza (Davies, 1982, p. 35).

Oxen were kept as beasts of burden, and were used for ploughing the land (Davies, 1982, p. 39). Different types of crops were noted in the written sources, and mention is made of corn, seeds, cornfields and harvesting (Davies, 1982, p. 38). The 9th‐century *Historia Brittonum* attributed to the monk Nennius refers to the winnowing of corn and, after reaping, sheaves of corn were stored in barns (Giles, 1841). Different grains were assigned different values, the most important being wheat which was used to bake white wheaten loaves, while barley was also used for bread, and oats were fed to horses (Davies, 1982, p. 38). Environmental sampling from archaeological sites across southwest Wales has shown that early medieval populations did indeed rely on oats and barley, as well as bread‐type wheats. For example, soil samples from Brownslade Barrow revealed charred plant remains including barley (*Hordeum* sp.), hulled wheat species (emmer/spelt, *Triticum dicoccum/spelta*), and domesticated oat species (*Avena* sp.). At the 9th–11th century settlement of South Hook in Pembrokeshire three early medieval corn dryers were found alongside evidence for the use of dredge—a mixed crop of oat and barley often used as fodder or for bread, oatcakes, or brewing—and the practice of malting (Carruthers, 2010), while quernstones—used for grinding corn—were recovered from the high‐status settlement of Dinas Powys in southeast Wales (Alcock, 1963; Davies, 1982, p. 35).

In addition to the cultivation of crops and the rearing of livestock, communities also relied on natural resources to supplement their diet. According to the 11th‐century *Life* of St David, the people of St Davids, Pembrokeshire were accustomed to collecting nuts, and at the site of Brownslade Barrow environmental samples indicate that hazelnuts and sloes were collected from hedgerows (Carruthers, 2012, p. 161). Fish, particularly riverine fish, were noted as a source of food; in southeast Wales, Saint Cadog, the abbot of the 5th/6th century monastery of Llancarfan, fought to secure the rights for his monastic communities to fish in the Usk and Neath rivers (Davies, 1982, p. 34). The late‐6th to late‐11th century Llandaff charters (*Liber Landavensis*) which record grants to the Church in and around southeast Wales, show that access to weirs and fishing rights could also be donated in addition to land (Davies, 1978). Giraldus Cambrensis wrote specifically about the availability of fish, referring to perch and eels in the northern lakes of Snowdonia and salmon, trout and greyling in the rivers of the south (Davies, 1982, p. 34). Bees were another utilised resource and an abundance of honey was seen as an indicator of fertile land; the 12th‐century *Life* of Saint Illtud praises the monastery at Llantwit Major for having an “abundance of flowers and honey” (Davies, 1982, p. 34). Law tracts record the value of swarms, hives, and wax suggesting that apiculture—rather than simply the collection of wild honey—was particularly important, most likely since honey was essential for making mead and Welsh ale, regarded as the drink of elites, poets, and heroes (Williams, 1980).

Access to agricultural land was not equal however, and those occupying the lower strata of society were expected to work the land for others. Evidence from the Llandaff charters demonstrates that much of the cultivatable land in southeast Wales was organised into estates, ranging from 40 to 6,000 acres, which was worked by tenant farmers and slaves (Davies, 1982, p. 42). The charters, and the Welsh lawbooks, such as the Book of Cyfnerth compiled in the 12th century, record that food renders were paid to landowners and territorial rulers (Charles‐Edwards, 2013, pp. 274, 280; Davies, 1982, p. 41). Food renders were given in the summer and winter, and consisted of three essential components; ale, bread and an accompaniment to the bread depending on the season, with a greater quantity of meat in winter and more dairy produce in summer. Honey was also given either to produce mead or as an accompaniment to the bread, while the winter render also had to include horse‐fodder (Charles‐Edwards, 2013, pp. 280–282). Some elite rulers during the 5th to 7th centuries AD also had the capacity to import luxury consumables (Campbell, 2007; Fulford, 1989; Thomas, 1959). For example, eastern Mediterranean amphorae produced in the Peloponnese, western Turkey, and Syria have been recovered from high‐status settlement sites in Wales, including Dinas Powys, and in Cornwall (Campbell, 2007). These vessels are believed to have contained wine, olive oil, and madder, while evidence for repaired amphorae suggests that some vessels could have carried dried goods (e.g., grain, nuts, spices) and not just liquids (Alcock, Stevenson, & Musson, 1995, p. 84).

Members of the monastic communities—particularly those resident at large estates in southeast Wales—also enjoyed a varied diet; for instance in his “Preface on Penance,” Gildas refers to the monastic diet consisting of a small portion of butter, cheese, milk, and buttermilk to drink alongside bread, broth, eggs, and garden vegetables, while the 5th/6th century monastic community at Llancarfan enjoyed fish and milk (Davies, 1982, p. 35). It is, therefore, apparent from the written sources that those who were in a position to demand the labor and produce of others could enjoy a rich and varied diet drawn from an agrarian economy, while some members of high‐status society had access to “exotic” foodstuffs. One particular advantage of investigating dietary resource consumption from the analysis of human remains excavated from early medieval cemetery populations is that the evidence will reflect the dietary resources consumed by all members of society, and will complement the elite perspective provided by written accounts.

## 2 Materials and methods

As part of this investigation, carbon, nitrogen and sulphur isotope analysis was undertaken on three cemetery populations from the Isle of Man (Peel Castle, Balladoole, Cronk keeillane) and six populations from Wales, including four cemeteries from southwest Wales (Brownslade Barrow, Porthclew, West Angle Bay, St Patrick's Chapel) and two cemeteries from southeast Wales (Llandough, Atlantic Trading Estate). Thirteen faunal samples were available from St Patrick's Chapel and analysed for carbon, nitrogen, and sulphur isotopes as a baseline study. The study sites from southwest Wales and the Isle of Man are located within 20 km of the Irish Sea, while in southeast Wales, Atlantic Trading Estate is situated near the mouth of the Cadoxton River which flows into the Bristol Channel, and the nearby site of Llandough overlooks the River Ely and is less than 20 km from the Severn Estuary. A summary of each site is provided below, and sampled skeletons date from the period between the early‐5th and early‐11th century AD.

### 2.1 Sampled cemetery sites: Isle of Man

#### 2.1.1 Peel Castle

**Figure 1:**
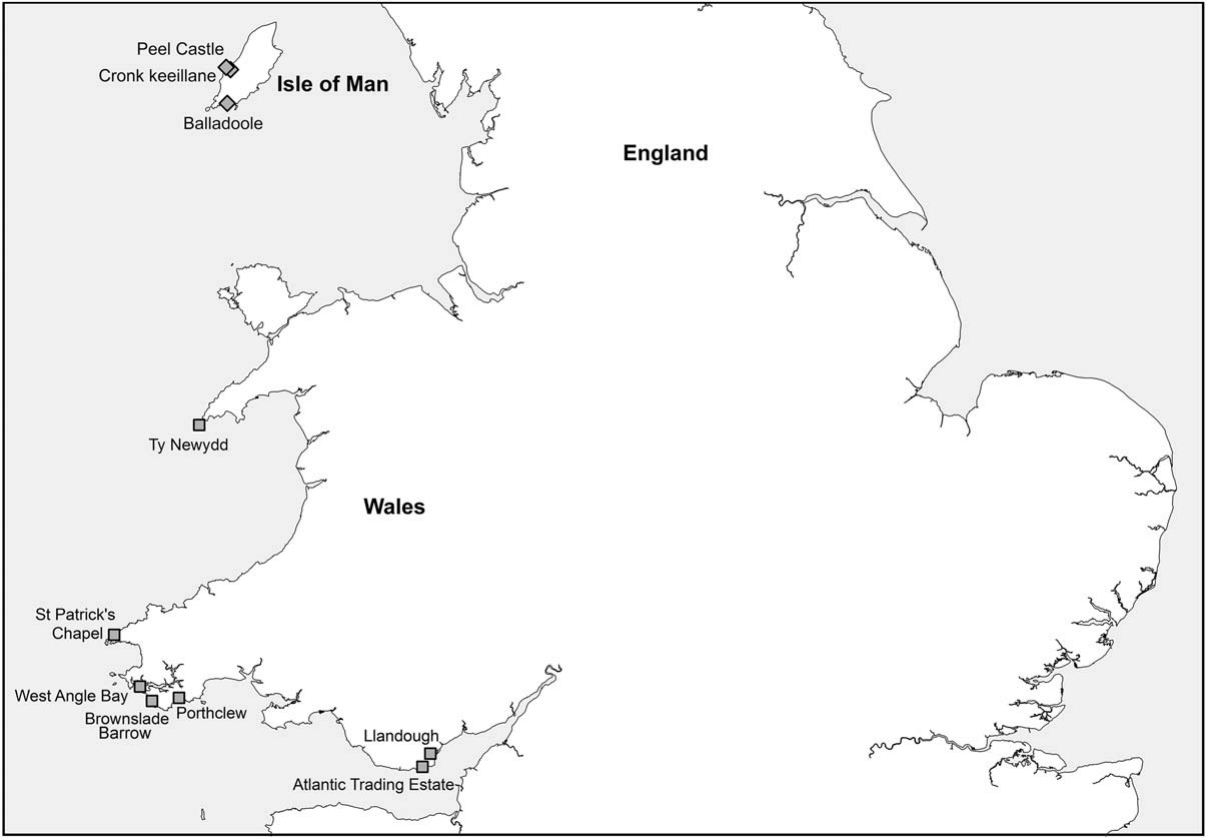
Map of Wales and the Isle of Man illustrating the location of the cemetery sites analysed by this study, and the location of Ty Newydd, Bardsey Island (Arnold, 1998)

St Patrick's Isle lies off the west coast of the Isle of Man (Figure 1), and is dominated by the ruins of Peel Castle and the 13th‐century cathedral of St German (Freke, 2002). Ecclesiastical activity on the isle dates back to the 10th−11th centuries AD, including St Patrick's Church and a small rectangular stone chapel known as a keeill (Freke, 2002). Excavations have been undertaken on the isle since the mid‐20th century, while in the 1980s a large‐scale systematic excavation conducted to the north of St German's Cathedral chancel revealed a cemetery of 327 burials consisting of simple dug graves and stone‐lined cist graves (Freke, 2002). Radiocarbon dating confirmed an early medieval date for the cemetery, with the earliest burials dating to AD 650–960 (2σ), and the latest dating to AD 1290–1440 (2σ). A total of 23 burials were identified as pre‐10th century or probable pre‐10th century date on the basis of their phase within the cemetery, available radiocarbon dates, datable coins, or their overall characteristics (e.g., stone‐lined cists) (Freke, 2002). Skeletal preservation was highly variable across the cemetery and many earlier burials were poorly preserved; Table 1 includes the osteological data from the original assessment (Rubin, 2002). Stable isotope analysis was undertaken on 10 skeletons believed to be of pre‐10th century date (Table 1).

**Table 1:** δ^13^C_VPDB_, δ^15^N_AIR_, and δ^34^S_VCDT_ values for the human skeletal samples with suitably preserved collagen

Sample^a^	Sex	Age	δ^13^C_VPDB_	δ^15^N_AIR_	% C	% N	C:N	δ^34^S_VCDT_	% S	C:S	N:S
ATE‐1	F	35–45	−20.8	9.8	42.4	15.0	3.3	15	0.2	594	180
ATE‐2	F	25–35	−20.9	11.4	41.7	14.8	3.2	14	0.2	696	211
ATE‐10	M	18–25	−20.6	11.8	38.4	13.0	3.4	10	0.2	488	142
ATE‐23	M	35–45	−20.7	11.0	40.7	14.2	3.3	12	0.2	571	171
ATE‐28	F	25–35	−20.7	10.0	44.4	44.4	3.3	14	0.2	592	507
ATE‐34	F	25–35	−20.6	10.2	44.3	15.8	3.2	11	0.2	621	190
ATE‐35	F	18−25	−21.0	9.5	44.1	15.5	3.3	14	0.2	534	161
ATE‐36	NA	13–17	−19.9	10.4	43.4	15.4	3.3	11	0.2	609	186
ATE‐40	M	35–45	−20.4	10.6	39.8	13.9	3.3	10	0.2	482	145
ATE‐41	M	35−45	−20.2	10.6	36.1	12.1	3.4	11	0.2	481	139
SP‐202	?	Adult	−20.9	10.0	24.1	7.8	3.6	16.5	0.1	536	16.5
SP‐205	M	>45	−21.2	10.5	39.3	12.6	3.6	16.6	0.3	400	16.6
SP‐206	F	35−45	−20.1	12.4	43.6	15.5	3.2	15.6	0.2	554	15.6
SP‐207	F	>45	−20.1	12.3	37.4	13.3	3.3	16.5	0.2	499	16.5
SP‐209	F	Adult	−20.8	12.1	41.9	14.3	3.4	15.7	0.2	486	15.7
SP‐210	F	18−25	−20.2	11.7	45.2	16.1	3.3	16	0.3	463	16
SP‐211	?	Adult	−20.6	11.0	29.6	10.1	3.4	17.3	0.3	272	17.3
SP‐213	NA	<1yr	−20.0	13.8	43.8	15.3	3.4	16.4	0.3	468	16.4
SP‐214	F	>45						14.8	0.2	314	14.8
SP‐216	F	18–25	−19.8	9.1	37.9	13.3	3.3	8	0.2	481	8
SP‐218	F	18–25	−20.5	11.9	38.7	13.6	3.3	15.9	0.2	469	15.9
SP‐226	?	Adult	−20.6	11.7	28.3	9.8	3.4	16.6	0.1	539	16.6
SP‐227	?	Adult	−20.6	10.4	35.5	12.2	3.4	15.0	0.2	526	15.0
SP‐230	?	Adult	−20.4	11.2	43.7	15.5	3.3	15.6	0.2	613	15.6
SP‐231	?	Adult	−20.5	11.5	39.8	14.0	3.3	14.8	0.2	505	14.8
SP‐232	?	Adult	−20.7	11.3	34.8	11.9	3.4	16.6	0.2	516	16.6
SP‐245	?	Adult	−20.2	12.1	27.4	9.5	3.4	16.3	0.2	487	16.3
BD 3	M	18–25	−20.7	11.6	42.3	14.8	3.3	17.6	0.2	752	226
BD 6	F	18–25	−21.1	12.5	38.4	13.1	3.5	17.1	0.2	569	166
BD 27	M	>45	−20.2	11.3	43.8	14.5	3.5				
CK 1226	M	Adult	−21.0	11.5	39.9	13.0	3.6				
CK 1581	F	25–35	−21.2	11.0	40.8	14.0	3.4	17.3	0.2	604	178
CK 1225	M	>45	−20.6	11.1	37.5	13.0	3.4				
CK 1234	M	35–45	−21.2	11.0	42.5	14.6	3.4	16.2	0.2	515	152
CK 1769	M	18–25	−21.0	11.0	38.5	12.7	3.6				
CK 1236	M	18‐25	−20.7	10.0	45.6	15.5	3.5				
BS 502A	F	>45	−20.5	10.9	39.1	13.0	3.5				
BS 508	F	25–35	−20.4	10.4	41.6	14.1	3.5	17.7	0.2	583	170
BS 511	F	25–35	−21.2	10.3	35.3	12.0	3.5	18.4	0.2	554	162
BS 514	NA	13–17	−20.7	10.7	34.9	11.9	3.5				
BS 517	M	>45	−20.3	10.9	41.5	14.1	3.5				
BS 528A	M	18–25	−20.4	11.0	41.7	14.1	3.5	16.7	0.2	556	161
BS 530	M	25–35	−20.1	10.8	44.3	15.4	3.4	17.9	0.2	563	168
BS 532	M	>45	−20.1	10.5	30.6	10.4	3.4				
BS 535	F	18–25	−20.1	10.3	41.1	14.4	3.4	17.8	0.1	843	252
BS 538	F	25–35	−20.0	10.4	40.4	14.1	3.4	17.5	0.2	567	170
LLD 002	F	25–35	−20.0	10.4	40.2	14.3	3.3	7.2	0.2	487	148
LLD 005	F	35–45	−20.7	10.5	33.1	10.9	3.5	7	0.2	384	108
LLD 010	M	35–45	−20.3	10.5	32.6	11.4	3.3	5.5	0.2	512	153
LLD 011	M	35–45	−20.5	9.7	32.0	10.5	3.5	7	0.2	533	150
LLD 016	F	25–35	−20.3	10.5	31.8	10.8	3.4	11	0.2	472	137
LLD 017	M	35–45	−20.6	11.3	34.7	11.7	3.4	9	0.2	441	127
LLD 029	F	25–35	−20.4	10.2	23.6	7.9	3.5	9	0.1	525	151
LLD 032	M	35–45	−20.0	10.1	27.9	9.1	3.6	7	0.2	413	115
LLD 072	F	35–45	−20.1	11.3	28.5	9.6	3.4	2.8	0.2	448	129
LLD 087	F	18–25	−20.2	12.4	34.5	11.7	3.4	7	0.2	437	127
LLD 088	M	25–35	−20.2	11.2	37.1	12.2	3.5	11	0.2	450	127
LLD 093	F	>45						9.1	0.2	473	172
LLD 098	M	35–45	−20.6	10.2	20.7	8.0	3.1	5.4	0.1	552	182
LLD 106	M	25–35	−19.8	9.7	36.4	12.5	3.4	6	0.2	443	130
LLD 110	NA	13–17	−21.0	10.0	21.9	7.2	3.5	9	0.1	417	117
LLD 112	F	18–25	−20.3	9.6	40.0	14.0	3.3	11	0.2	534	160
LLD 126	M	35–45	−20.2	9.9	26.8	10.5	3.5	1.2	0.2	420	141
LLD 206	NA	7–12	−20.2	10.0	37.4	13.0	3.3				
LLD 212	NA	7–12	−20.0	9.5	33.4	10.9	3.5	6	0.2	445	125
LLD 222	F	25–35	−20.2	10.7	39.0	13.2	3.4	6	0.2	495	143
LLD 333	M	>45	−19.9	11.5	40.0	13.6	3.4	6	0.2	464	135
LLD 362	M	>45	−20.3	10.6	20.7	8.2	3.0	4.3	0.1	460	156
LLD 409	M	35–45						8	0.2	319	116
LLD 415	F	18–25	−20.4	11.3	18.5	7.7	2.9	6.3	0.1	449	160
LLD 470	NA	13–17	−19.4	9.6	41.6	14.8	3.3	10	0.2	554	169
LLD 474	F	25–35	−20.8	9.1	27.4	12.1	2.9	8.6	0.2	385	146
LLD 517	F	35–45	−20.0	10.2	31.8	10.9	3.4	10	0.2	472	139
LLD 542	M	>45	−20.8	11.3	27.4	9.1	3.5	12	0.1	523	149
LLD 566	M	>45	−19.9	9.8	23.1	7.7	3.4	10	0.1	473	136
LLD 864	F	35–45	−19.7	10.7	41.0	14.2	3.3	12	0.2	576	171
LLD 868	F	25–35	−20.2	10.3	26.0	8.9	3.4	10	0.1	533	157
LLD 877	F	35–45	−20.2	9.4	26.3	8.9	3.4	11	0.2	468	136
LLD 972	F	18–25	−19.8	10.3	39.2	13.8	3.3	9	0.2	498	150
PC 01	M	35–45	−20.8	10.0	42.0	14.6	2.9				
PC 02	M	35–45	−20.9	11.3	41.4	13.8	3.0				
PC 03	NA	7–12	−20.8	10.2	42.8	15.0	2.9				
PC 04	NA	7–12	−20.9	9.9	42.4	14.8	2.9				
PC 05	F	35–45	−21.0	10.1	40.8	13.9	2.9				
WAB 059	?	18–25	−20.6	12.3	38.9	13.0	3.0				
PE 1118	F	Adult	−20.8	11.7	41.0	14.0	2.9				
PE 1155	M	Adult	−20.6	11.7	33.0	11.2	3.0				
PE 595/597	?	Adult	−20.7	11.9	36.9	12.5	3.0				
PE 587	?	Adult	−20.0	11.6	38.6	13.4	2.9				

a Sites are represented by the following codes: CK = Cronk keeillane, BD = Balladoole, PE = Peel Castle; ATE = Atlantic Trading Estate, LLD = Llandough, BS = Brownslade, PC = Porthclew, WAB = West Angle Bay, SP = St Patrick's Chapel. Sex categories are M =Male, F = Female, NA = Non‐adult, ?=Undetermined.

#### 2.1.2 Balladoole

The cemetery of Balladoole occupies a small hillock (“Chapel Hill”) on the southeast coast of the Isle of Man (Figure 1). The site was excavated in 1945 by Professor Gerhard Bersu and is most well‐known for the discovery of a boat burial believed to date to the Viking Age (Bersu & Wilson, 1966; Wilson, 2008). Below, and thus predating the boat burial, was a cemetery of stone‐lined cist burials, which have been confirmed as dating to the 4th–7th century AD through radiocarbon dating (Fox, pers.comm). According to the original excavation report by Bersu, burials were oriented east–west (head to the west end), without grave goods, and individuals were buried supine with their arms by their sides and hands placed on the pelvis (Bersu & Wilson, 1966). A reassessment of the skeletal remains was undertaken by KH, which identified both adults and non‐adults (<18 years of age) (Bunting & Verity, 1960; Hemer, 2010). In total, 28 individuals were identified, 18 of whom could be associated with the *in situ* burials recorded on Bersu's excavation plan, while a minimum of 10 individuals were identified as mixed/comingled. Four individuals were analysed for carbon and nitrogen isotope analysis and two were sampled for sulphur analysis (Table 1).

#### 2.1.3 Cronk keeillane

The cemetery of Cronk keeillane (“Mound of the little church”) lies en‐route to the town of Peel on the west side of the Isle of Man (Figure 1) (Kermode, 1926). Very little is known about the cemetery itself asides from that recorded in antiquarian accounts from the late‐19th and early‐20th centuries (Barnwell, 1868; Kermode, 1926; Oswald, 1860). The antiquarian reports refer to a cemetery east of a small barrow, where graves of various sizes were arranged in a parallel fashion (Oswald, 1860). In 1925, excavation revealed the remains of a keeill, and a cross‐inscribed stone dated by P.M.C. Kermode to the 6th century AD (Kermode, 1926). During the 1980s, the cemetery was excavated and human skeletal remains were curated by Manx National Heritage, Douglas, however, the actual excavation report was never published. Recent radiocarbon dating has confirmed an early medieval date for five skeletons; four date to AD 564–654 (2σ) while another dates to AD 656–770 (2σ) (Hemer, 2010, 2012; Hemer, Evans, Chenery, & Lamb, 2014). An assessment of the human remains by KH identified 16 adults and two non‐adults; seven adult individuals were selected for isotopic analysis (Table 1).

### 2.2 Sampled cemetery sites: Wales

#### 2.2.1 Llandough (southeast Wales)

The village of Llandough overlooks the River Ely, approximately 3.5 km from the modern‐day city of Cardiff (Holbrook & Thomas, 2005, p. 1) (Figure 1). Within the village, the church of St Dochdwy is believed to occupy the site of a major early medieval monastery recorded in the Llandaff charters (Holbrook & Thomas, 2005, p. 1). An area of 0.22 ha was excavated in 1994, and revealed 1,026 burials making it the largest early medieval cemetery excavated in Wales to date. Activity around the later church of St Dochdwy dates back to the Iron Age, while a Roman villa was constructed during the 2nd century AD and was occupied until the early‐4th century AD (Holbrook & Thomas, 2005, p. 2). Radiocarbon dating confirmed that burial at the site had commenced by the period AD 370–640, and continued until around the 11th century AD (Holbrook & Thomas, 2005, p. 88). Most burials were single, extended inhumations oriented east‐west (head at the west end), however, there were examples of multiple burials as well as distinctive rites including flexed, prone, crouched and seated burials. There was a dearth of stone‐lined cist burials, but a number of graves contained small corroded iron fragments and black organic staining suggesting the use of wooden coffins (Holbrook & Thomas, 2005, pp. 26–27). A total of 814 articulated skeletons and 212 disarticulated bone groups were excavated and represent the remains of males, females, and non‐adults (Loe, 2003). Dietary stable isotope analysis was undertaken on 36 individuals from the cemetery (Table 1).

#### 2.2.2 Atlantic Trading Estate (southeast Wales)

The site of Atlantic Trading Estate once consisted of a low promontory with the Bristol Channel to the south, and a sheltered bay and Barry Island to the northwest (Figure 1). The present course of the Cadoxton River also runs through the site which consists of sand dunes and marshland (Price, 1996). It is thought that the promontory was linked to Barry Island via a causeway, which was part of a pilgrim route to the chapel of St Barruc (Price, 1996). Excavation at Atlantic Trading Estate during the 1980s revealed an early medieval cemetery consisting of east–west (head to the west end) oriented burials cut into the sand dune. A total of 45 graves were excavated, all of which were in keeping with the funerary rites of this period, including simple sand‐dug graves, the use of shrouds, stone‐lined cist burials and occasionally, the use of wooden planks (Price, 1996). Osteological analysis of the skeletal remains by Loe (2003) revealed a mixed cemetery population consisting of males, females, and non‐adults. Radiocarbon dates confirm that the cemetery was in use for burial between the late‐4th and early‐6th centuries AD (Price, 1996). A total of 10 skeletons, including nine adults and one non‐adult were sampled for stable isotope analysis (Table 1).

#### 2.2.3 Brownslade Barrow (southwest Wales)

Brownslade Barrow is a Scheduled Ancient Monument situated in the Castlemartin Parish on the south Pembrokeshire coast and lies approximately 1.6 km from the sea in an area of wind‐blown sand (Figure 1) (Groom et al., 2012). Considerable damage to the barrow by badger activity was observed in 2001, and a number of burials were disturbed (Groom et al., 2012, pp. 136–137). In response, the site was surveyed in 2002 and excavated in 2003 by Dyfed Archaeological Trust. The excavation revealed a total of 32 burials containing the remains of 52 well‐preserved skeletons. Radiocarbon dates confirm that the cemetery was in use between AD 430 to AD 1020 (2σ), with a considerable phase of burial activity between the mid‐7th to late‐9th century AD (Groom et al., 2012). The east–west (head to the west end) orientation of the burials, the form of the burials including simple earth‐dug graves and stone‐lined cist burials, and the absence of grave provisions, were consistent with early Christian tradition. Analysis of the skeletal remains revealed a mixed community of adult males, females, and nonadults (Hemer, 2010). Carbon, nitrogen, and sulphur analysis was undertaken on 10 individuals excavated from the site (Table 1).

#### 2.2.4 Porthclew (southwest Wales)

The cemetery of Porthclew lies near the ruined remains of Porthclew chapel, close to the beach of Freshwater East in Pembrokeshire (Schlee, 2009, p 1) (Figure 1). Human remains were uncovered in the area during the construction of a house in 1964, and the laying of an electricity cable in 1999. In 2002, Dyfed Archaeological Trust undertook a geophysical survey of the site, which was later excavated in 2008 (Schlee, 2009, pp. 1–3). Six trenches were excavated across the site, four of which revealed human remains, and simple earth‐dug graves and stone‐lined cist graves. Radiocarbon dates for five burials confirmed that the site was in use during the early medieval period, with the earliest burial dating to AD 430–610 (2σ), and the latest dating to AD 680–900 (2σ). Eight individuals, including five adults and three non‐adults, were recorded during the osteological analysis by KH; five were sampled for stable isotope analysis (Table 1).

#### 2.2.5 West Angle Bay (southwest Wales)

The cemetery of West Angle Bay is located on a cliff overlooking the Milford Haven Estuary in Pembrokeshire (Schlee & Ludlow, 2012, pp. 167–177) (Figure 1). The cemetery consists of an egg‐shaped earthwork enclosure defined by a stone bank, which is contained within a larger rectangular enclosure. The northern edge of the rectangular enclosure has been lost from the edge of the cliff due to coastal erosion, and it was this erosion that exposed burials and human remains in 1997 (Schlee & Ludlow, 2012, pp. 171–176). Dyfed Archaeological Trust were commissioned to excavate part of the site in 2005 and 2006; 15 burials were identified in and around the earthwork and enclosure. The earliest burial was dated to AD 650–780 (2σ) while the site continued as a cemetery until AD 890–1120 (2σ) (Schlee & Ludlow, 2012, pp. 171, 177). Preservation of the skeletal remains was very poor and as a consequence it was only possible to estimate a minimum number of 25 individuals, amongst which were a large proportion of non‐adults (Schlee & Ludlow, 2012, p. 173). Owing to such poor skeletal preservation it was only possible to sample three individuals for stable isotope analysis (Table 1).

#### 2.2.6 St Patrick's Chapel (southwest Wales)

Excavation of the early medieval cemetery of St Patrick's Chapel, which overlooks Whitesands Bay in Pembrokeshire, begun in 2014 following the exposure of human remains during severe winter storms (Figure 1). Work on the project is on‐going, however more than 50 burials have been excavated so far and these predate the foundation of a later medieval stone chapel. Radiocarbon dates confirm that the cemetery was in use for burial from at least the 7th century AD. The cemetery population consists of males, females and non‐adults, and there is a predominant use of stone‐lined cists and evidence for the use of crossinscribed grave markers (Murphy, Shiner, Wilson, & Hemer, 2016). Stable isotope analysis was undertaken on 17 human skeletons and 13 faunal samples collected from the cemetery site (Tables 1 and 2).

**Table 2:** δ^13^C_VPDB,_ δ^15^N_AIR_, and δ^34^S_VCDT_ values for the faunal samples from St Patrick's Chapel with suitably preserved collagen

Sample	Taxon	Bone	δ^13^C_VPDB_	δ^15^N_AIR_	% C	% N	C:N	δ^34^S_VCDT_	% S^a^	C:S	N:S
T1 [13]	sheep	leg	−23.1	8.2	41.6	14.3	3.4	4.7	0.2	498	147
STPC'15 [97]	sheep	leg	−22.2	7.8	38.0	13.0	3.4	15.1	0.2	550	161
STPC'15 (w/236)	sheep	tibia?	−21.9	7.4	43.0	14.8	3.4	17.0	0.2	562	166
73	sheep	?	−22.4	7.2	39.5	13.7	3.4	16.9	0.2	554	165
T1 [13]	sheep/goat	?	−21.2	6.5	27.5	9.4	3.4	17.3	0.2	438	128
T1[T1]	sheep/goat	leg	−22.6	9.1	24.7	8.3	3.5	18.4	0.1	473	136
#103	sheep/goat	leg	−21.3	6.3	28.8	10.0	3.4	17.4	0.1	567	168
73	sheep/goat	shoulder?	−22.1	6.3	29.3	10.1	3.4	17.2	0.2	513	151
STPC'15 (w/236)	pig	mandible	−22.0	9.8	26.7	9.1	3.4	17.8	0.2	456	133
STPC'15 [73]	cow	?	−22.0	6.7	26.8	8.8	3.5	17.1	0.1	501	142
STPC'14 (T1) [13]	cow	leg	−22.3	6.7	29.6	10.2	3.4	17.6	0.2	400	118
STPC'15	cow	?	−21.8	7.2	29.1	10.0	3.4	17.5	0.2	443	130
STPC'14 (nr 216)	cow	? foot	−22.1	6.4	36.6	12.8	3.4	17.7	0.2	593	177
**Mean**			−**22.1**	**7.3**				**16.3**			
**1σ**			** 0.5**	**1.1**				**3.6**			

a Calculated %S.

### 2.3 Principles of palaeodietary reconstruction

The principles of palaeodietary reconstruction and the use of carbon and nitrogen isotope analysis are well‐established and discussed at length elsewhere (e.g., Ambrose, 1990; Lee‐Thorp, 2008; Müldner & Richards, 2005; Pate, 1994; Sealy, 2001), and therefore, it is only necessary to provide a brief overview here. The use of carbon and nitrogen isotopes for palaeodietary reconstruction relies on the principle that carbon and nitrogen isotopes present in consumed foods (i.e., dietary proteins, carbohydrates, and lipids) are incorporated into body tissues (e.g., bone collagen, dentine, and hair). Since bone remodels during life, the isotope composition of bone collagen from the rib reflects the isotope profile of the foods consumed (predominantly the protein) during the last two to five years of life (Cox & Sealy, 1997; Sealy, Armstrong, & Schrire, 1995). Carbon isotope values recorded for bone collagen will reflect the δ^13^C values of consumed plants and animals, and can be used to distinguish between C_3_ and C_4_ plants which follow two different photosynthetic pathways (O'Leary, 1988; Pate, 1994; Schwarz & Schoeninger, 1991). Since C_4_ plants are not indigenous to the British Isles or northern Europe, the application of carbon isotope analysis to populations from Britain and Ireland is usually focused on distinguishing between the consumption of marine‐sourced and terrestrially‐sourced foods. It is accepted that a diet reliant entirely on terrestrial resources, including C_3_ plants, would result in a collagen δ^13^C value of −20 ±1‰, while a diet comprising entirely of marine protein would be reflected by a δ^13^C value of −12 ±1‰ (Richards, Fuller, & Molleson, 2006, p. 123).

Nitrogen isotope analysis is applied in conjunction with carbon isotope analysis to further identify the consumption of animal protein, and to distinguish between terrestrial and marine resources. Nitrogen incorporated into body tissues is obtained from the breakdown of amino acids from the protein portion of the diet during biochemical processes (e.g., protein metabolism), and undergo a process of fractionation which occurs at each trophic level (DeNiro & Epstein, 1981). While the amount of ^15^N fractionated during metabolic processes can be influenced by various factors (e.g., physiological adaptations, environmental habitats, physiological stress), an increase of 3–4‰ in the δ^15^N value of the consumer relative to its source of dietary protein are observed at each successive trophic level (e.g., between carnivores and herbivores) (Ambrose, 1991, 2000; Fuller et al., 2005; Hedges & Reynard, 2007; Schoeninger & DeNiro, 1984; Sponheimer et al., 2003). Such a trophic shift can also be observed in breastfeeding infants who are consuming maternal milk (Fogel, Tuross, Johnson, & Miller, 1997; Fuller et al., 2006; Jenkins, Partridge, Stephenson, Farley, & Robbins, 2001). Nitrogen isotope analysis can also be used to identify the consumption of aquatic resources since the longer food chains within these environments increases the number of trophic levels resulting in significantly higher δ^15^N values. The consumption of marine protein will also result in higher δ^13^C values (Pate, 1994; Schwarz & Schoeninger, 1991) while freshwater fish consumption can appear to have an overall “terrestrial profile” since freshwater fish have δ^13^C values comparable to terrestrial species (Schwarz & Schoeninger, 1991, p. 304).

A more recent addition to the investigation of palaeodiet is the use of sulphur isotopes, which are incorporated into bone collagen from the essential amino acid, methionine (Nehlich, 2015; Richards et al., 2001, pp. 1–17). Methionine is synthesised by plants from inorganic sulphur leached from the underlying geology into the soil, and is passed on to animals and fish as dietary methionine with minimal fractionation (<1‰) at each trophic level (Nehlich, Boric, Stefanovic, & Richards, 2010, p. 1131; Nehlich et al., 2011, pp. 4965–4966; Trust & Fry, 1992). As such, δ^34^S of bone collagen in humans relates to the sulphur isotope ratios of the soil and plants available in a particular region (Nehlich, 2015, pp. 3–5; Nehlich et al., 2011, p. 4967), and knowledge of the sulphur isotope composition of the local geology and water sources can also be used to identify non‐local individuals (Nehlich et al., 2010, p. 1132). Sulphur isotope analysis is used to distinguish between consumers of marine and terrestrial resources since terrestrial organisms are said to have low, but more variable, sulphur isotope values ranging from −5‰ to +10‰ (Krouse, 1980), while marine organisms cluster around a δ^34^S value of c. +20‰, which is the value of marine sulphate (Richards et al., 2001, p. 186). Freshwater systems can demonstrate considerable variability with δ^34^S values ranging from −22‰ to 20‰ (Richards et al., 2001, p. 186), and therefore to identify the consumption of freshwater fish reliably requires knowledge of local baseline δ^34^S values from riverine sources (Nehlich et al., 2011; Privat, O'Connell, & Hedges, 2007). Soil values for coastal regions often exhibit δ^34^S values of +20‰ since marine sulphates are introduced to the coastal ecosystem from sea‐spray and coastal precipitation (Lamb, Melissa, Ives, & Evans, 2012, p. 766; Nehlich, 2015, pp. 1–17; Richards et al., 2001, p. 186). As a result, inhabitants living within 20 km of the sea would be expected to have much higher δ^34^S values than populations living further inland, and therefore to confirm the consumption of marine resources it is also necessary to consider carbon and nitrogen data.

### 2.4 Laboratory methods

Carbon and nitrogen isotope analysis was undertaken on nine cemetery populations dating to the early medieval period from Wales and the Isle of Man (Figure 1). In total, 102 individuals—including adult males, females, and non‐adults were sampled (Table 1). While preference was given to the analysis of collagen from rib bone—since this limited any damage to other, intact skeletal elements—in some instances, bone preservation was so poor that it was necessary to extract collagen from the primary dentine portion of a tooth.

Collagen was extracted from fragments of rib bone or tooth dentine according to the method of Longin (1971). For the collection of dentine, each tooth was cut in half using a diamond‐edged rotary dental saw, and any secondary dentine was removed using a tungsten carbide dental burr. Remaining primary dentine was separated from the enamel surface and collected for analysis; the remaining enamel was retained for strontium and oxygen analysis (Hemer, Evans, Chenery, & Lamb, 2013; Hemer et al., 2014). Approximately 0.5–1 g of clean rib bone or 30–100 mg of dentine was immersed in 8 ml of cold 0.5 M HCL to demineralise. Following the removal of the solution, the sample was rinsed, and then solubilised in a solution of ph3 HCL on a hot block for 48 h at 70 °C. A 8 μm Ezzee filter was used to remove any solids from the solution in advance of freeze drying. As all samples satisfied the quality criteria outlined by DeNiro (1985) and Nehlich and Richards (2009), molecular weight filters were not used as in this case it was not deemed necessary. A sample of collagen was weighed into individual tin capsules and analysed in duplicate. Those prepared for sulphur received additional V_2_O_5_ in each capsule to aid combustion of the sulphur. Analysis was by Continuous Flow Isotope Ratio Mass Spectrometry using an Elemental Analyser (Flash/EA) coupled to a ThermoFinnegan DeltaPlus XL isotope ratio mass spectrometer via a ConFlo III interface. δ^13^C, δ^15^N, and δ^34^S ratios are expressed using the delta notation (δ) in parts per thousand (‰) relative to the international standards (VPDB, AIR, and VCDT) as follows: δ (‰)= (*R* sample/*R* standard)−1) × 1,000. δ^13^C and δ^15^N values were calibrated using the in‐house M1360p reference material (commercially available gelatine) which has an expected δ^13^C value of −20.32‰ (calibrated against IAEA‐CH‐7 and NBS22) and δ^15^N value of +8.12‰ (calibrated against IAEA‐N‐1 and IAEA‐N‐2). The average standard deviation of the duplicates was ± 0.13‰ for δ^13^C and ± 0.08‰ for δ^15^N. Samples for sulphur analysis were run in duplicate, and δ^34^S values were standardised using an in‐house standard, BROC‐2 (freeze‐dried broccoli), which has expected delta value of 11.67‰ (calibrated against IAEA‐S‐1 and IAEA‐S‐2). The average standard deviation of the duplicates was ± 0.20‰ for δ^34^S values and for weight % S was ± 0.005%. The 1σ reproducibility for mass spectrometry controls for these analyses was better than δ^15^N = ± 0.1‰, δ^13^C = ± 0.1‰, and δ^34^S = ± 0.2‰, respectively.

## 3 Results

**Figure 2:**
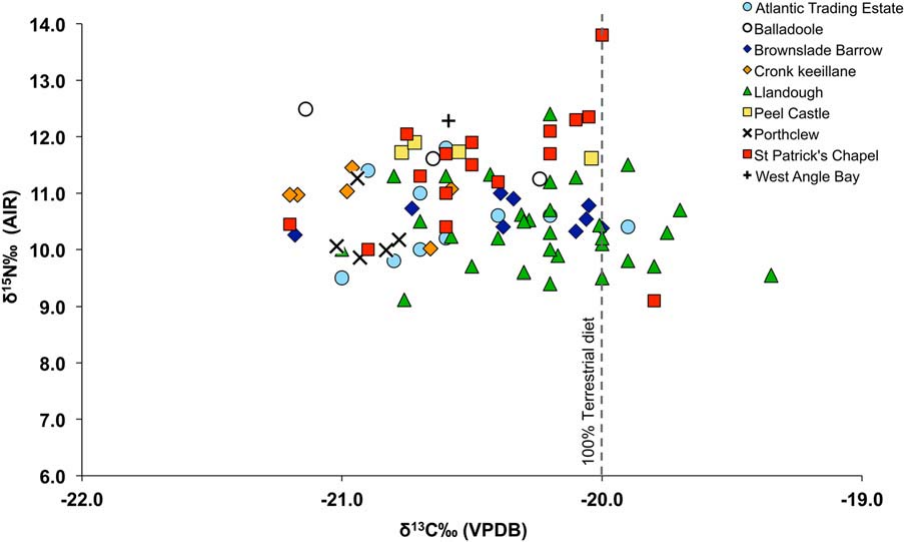
δ^13^C_VPDB_ versus δ^15^N_AIR_ results for the human skeletal remains analysed by this study

In total, 115 samples of collagen were analysed for carbon and nitrogen isotope analysis (Tables 1 and 2; Figure 2), and of these, 99 samples were considered to have C:N ratios that correspond to suitably preserved collagen (C:N 2.9‐3‐6) according to DeNiro (1985) (Tables 1 and 2). Many of the lowest C:N ratios (∼2.8/2.9) were recorded for samples of dentinal collagen, which was only used when bone was unavailable or poorly preserved. The low C:N ratios for many of the dentinal collagen samples therefore seems to correlate with poor skeletal preservation overall. The results for 16 samples were therefore excluded since the C:N ratios were outside the acceptable range (<2.9 or >3.6).

The carbon values for the human sample (*n* = 86) range from −19.4‰ to −21.2‰, with a mean of −20.4 ±0.4‰ (1σ). The nitrogen values for the sample (*n* = 86) range from 9.1‰ to 13.8‰ with a mean of 10.8 ± 0.9‰ (1σ) (Table 3). Of the total study sample, sulphur isotope analysis was undertaken on 69 individuals from six sites, and 13 faunal samples from St Patrick's Chapel (Tables 2 and 4). 66 samples had atomic C:S ratios between 300 and 900 and atomic N:S between 100 and 200 and were thus considered to represent well‐preserved collagen; three samples were excluded from further analysis (Nehlich & Richards, 2009). The human sulphur isotope values range from 1.2‰ to 18.4‰ with an overall average value of 11.6 ± 4.5‰ (1σ, n = 66) and the sulphur isotope data from the faunal material from St Patrick's Chapel range from 4.7‰ to 18.4‰ with an overall average value of 16.3 ± 3.6‰, (1σ, n = 13). Table 4 shows the mean sulphur isotope values for each site.

**Table 3:** Mean δ^13^C_VPDB_ and δ^15^N_AIR_ values for each site and each geographic region (human skeletal sample only, excluding 16 samples with C:N ratios <2.9 or > 3.6)

Region	Regional mean δ^13^C	1σ	Regional mean δ^15^N	1σ	Site	*n*=	Site mean δ^13^C	1σ	Site mean δ^15^N	1σ
Isle of Man	−20.7	0.4	11.4	0.6	Balladoole	3	−20.7	0.5	11.8	0.6
					Peel Castle	4	−20.5	0.3	11.7	0.1
					Cronk keeillane	6	−20.9	0.3	10.9	0.5
Southwest Wales	−20.5	0.4	11.0	1.0	Brownslade Barrow	10	−20.4	0.4	10.6	0.3
					St Patrick's Chapel	16	−20.4	0.4	11.4	1.1
					Porthclew	5	−20.9	0.1	10.3	0.5
					West Angle Bay	1	−20.6	0	12.3	0
Southeast Wales	−20.3	0.4	10.4	0.7	Llandough	31	−20.2	0.4	10.4	0.7
					Atlantic Trading Estate	10	−20.6	0.3	10.5	0.7
					Total	86	−20.4	0.4	10.8	0.9
					Minimum value		−21.2		9.1	
					Maximum value		−19.4		13.8	

**Table 4:** Mean δ^34^S_VCDT_ values for each site and each geographic region (human skeletal sample only)

Region	Regional mean δ^34^S	1σ	Site	*n*=	Site mean δ^34^S	1σ
Isle of Man	17.1	0.6	Balladoole	2	17.4	0.4
			Cronk keeillane	2	16.8	0.8
Southwest Wales	16.1	2.1	Brownslade Barrow	6	17.7	0.6
			St Patrick's Chapel	15	15.5	2.2
Southeast Wales	8.8	3.0	Llandough	32	7.9	2.6
			Atlantic Trading Estate	9	12.0	1.9
			Total	66	11.6	4.5
			Minimum value		1.2	
			Maximum value		18.4	

### 3.1 Comparison between age and sex

It was possible to compare carbon, nitrogen and sulphur values for males and females from the study sample to explore whether any differential access to dietary resources existed on the basis of sex. Statistical analysis was undertaken on data obtained for bone collagen only to compare the dietary profiles of adult individuals of known biological sex; an alpha level of .05 was used for all statistical tests. Mean carbon, nitrogen and sulphur isotope values according to sex are noted in Table 5. No statistically significant difference was observed between the mean carbon isotope values for males (mean male δ^13^C = −20.4 ±0.4‰, *n* = 26/60) and females (mean female δ^13^C = −20.4‰, ±0.4‰, *n* = 34/60; Independent *t* test: *t*=−0.146 df = 58, *p* = .884). Comparison between the mean nitrogen isotope values of males (male mean δ^15^N = 10.8 ±0.6‰, *n* = 26/60) and females (female mean δ^15^N = 10.7 ±1‰, *n* = 34/60) also revealed no statistically significant difference (Independent *t* test: *t* = 0.476, df = 56, *p* = .636). The mean sulphur isotope values for males was 10.0 ±4.7‰ (*n* = 22/66), while the mean sulphur values for females was 12.0 ±4.3‰ (*n* = 32/66) (Table 5); no statistically significant difference was reported between these mean values even though the female mean is slightly higher (Independent *t* test: *t*=−1.611, df = 52, *p* = .113). That there was no significant difference between the carbon, nitrogen or sulphur isotope values for males and females suggests that there was no differential access to dietary resources on the basis of sex amongst these populations.

**Table 5:** Mean δ^13^C_VPBD_, δ^15^N_AIR_, and δ^34^S_VCDT_ values for sexed adult individuals sampled for bone collagen only

Sex	*n*=	Mean δ^13^C	1σ	Mean δ^15^N	1σ	Mean δ^34^S	1σ
Male	26	−20.4	0.4	10.8	0.6	10.0 (*n*=22)	4.7
Female	34	−20.4	0.4	10.7	1	12.0 (*n*=32)	4.3

Since dentinal collagen forms during childhood and does not remodel, it provides a dietary signature for the individual at the time of tooth formation. As most samples of primary dentine were retrieved from the second permanent molars, the carbon and nitrogen isotope values will indicate the individual's childhood diet between three and seven years of age (Beaumont, Gledhill, Lee‐Thorp, & Montgomery, 2013; Hillson, 1996). The analysis of bone and dentinal collagen provides the opportunity to compare the diets of adult individuals with the diets of non‐adults, that is, individuals aged biologically below the age of 18. Mean carbon and nitrogen isotope values reflecting the dietary profiles of adults and non‐adults are presented in Table 6. Comparison between the mean carbon and nitrogen isotope values revealed no statistically significant difference (mean δ^13^C Independent *t* test: *t* = 0.167, df = 84, *p* = .868; mean δ^15^N values Independent *t* test: *t*=−0.413, df = 21.6, *p* = .684) between adults and non‐adults. Nonetheless, it is worth noting that the highest nitrogen isotope value (δ^15^N = 13.8‰) was recorded for an infant (SP 213) from St Patrick's Chapel. It seems plausible that this individual's elevated nitrogen isotope value—which is almost +3‰ higher than the mean nitrogen isotope value for the sampled population—indicates that this infant was breastfeeding, or had only recently begun weaning at the time of death and was thus at a higher trophic position relative to its mother.

**Table 6:** Mean δ^13^C_VPDB_ and δ^15^N_AIR_ values for the study sample according to age; the “<18 years” category includes values for samples from biological non‐adults and the values obtained from samples of dentinal collagen

Age	*n*=	Mean δ^13^C	1σ	Mean δ^15^N	1σ
Adult	68	−20.4	0.4	10.8	0.8
<18	18	−20.5	0.5	10.8	1.1

### 3.2 Comparison between sites and geographic regions

Since the study included cemetery populations from three different regions of western Britain (Isle of Man, southwest Wales, southeast Wales), consideration was given to the possibility that geographic location may have influenced access to, or preference for, certain dietary resources; for instance, the use of marine resources by the island populations of Man. The mean carbon and nitrogen isotope values for each region are: Isle of Man (mean δ^13^C = −20.7 ±0.4‰, δ^15^N = 11.4 ±0.6‰, *n* = 13/86), southwest Wales (mean δ^13^C = −20.5 ±0.4‰, δ^15^N = 11.0±1‰, *n* = 32/86) and southeast Wales (mean δ^13^C = − 20.3 ±0.4‰, δ^15^N = 10.4 ±0.7‰, *n* = 41/86) (Table 3). Statistical analysis confirmed a significant difference at the *p* < .05 level by One‐Way ANOVA between the mean carbon values (One‐way ANOVA: *F*(2,83)=7.25, *p* = .001) and mean nitrogen values (One‐Way ANOVA: *F*(2,83)=8.99, *p* = .00) for the three geographical regions. *Post hoc* comparisons using Bonferroni test revealed no significant difference between the mean nitrogen isotope values for the Isle of Man and southwest Wales, but that a difference did exist between the mean nitrogen isotope values for the Isle of Man/southwest Wales versus southeast Wales. A *post hoc* comparison using Bonferroni test also revealed that the mean carbon isotope values for the Isle of Man did differ significantly to southeast Wales but not to the mean carbon isotope values for southwest Wales.

Consideration of the mean sulphur isotope values for each geographic region also revealed a difference between the sites situated on/near the west coast of Wales, and those sites from southeast Wales. The mean sulphur isotope values according to each region are: Isle of Man (mean δ^34^S = 17.1 ±0.6‰, *n* = 4/66), southwest Wales (mean δ^34^S = 16.1 ±2.1‰, *n* = 21/66), and southeast Wales (mean δ^34^S = 8.8 ±3‰, *n* = 41/66) (Table 4). A statistically significant difference between the three regions was determined (One‐way ANOVA: *F*(2, 63) = 60.76, *p* = .000). Bonferroni's *post hoc* comparison of the three regions indicates that the mean sulphur isotope value for the Isle of Man was not statistically different to the mean sulphur isotope value for southwest Wales, while the mean sulphur isotope value for southeast Wales did differ significantly from both the mean sulphur isotope values for the Isle of Man and southwest Wales. This suggests that the populations from the southwest coast of Wales and the Isle of Man have sulphur isotope values that are more similar to each other than to the sulphur isotope values reported for the populations from southeast Wales.

## 4 Discussion

**Figure 3:**
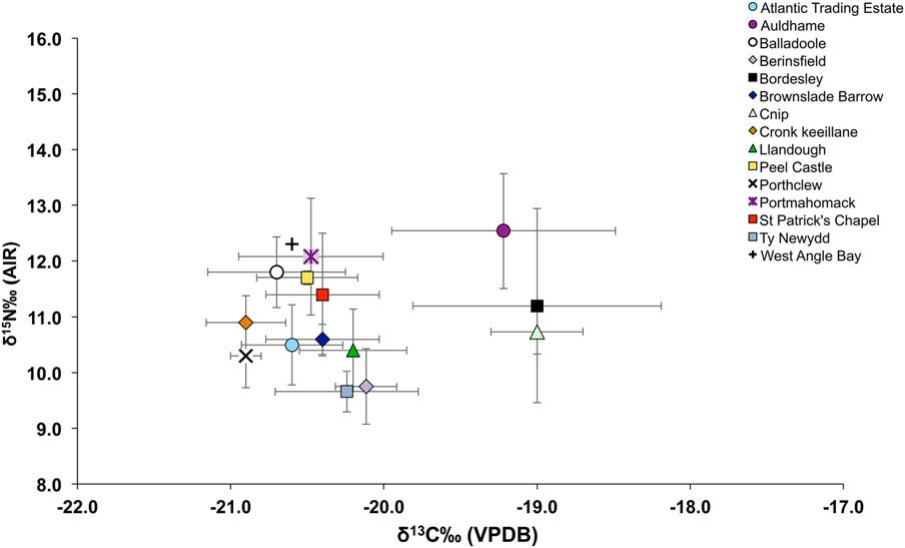
δ^13^C_VPDB_ versus δ^15^N_AIR_ values for the study sites compared with data drawn from published studies of medieval coastal and inland populations from the British Isles (Arnold, 1998; Curtis‐Summers et al., 2014; Lamb et al., 2012; Privat & O'Connell, 2001; Richards et al., 2001)

The carbon and nitrogen results obtained for the study sample are consistent with a terrestrial diet since the majority of the sample have carbon isotope values that are below −20 ±1‰, which is believed to be the threshold for a 100% terrestrial diet (Richards et al., 2006, p. 123) (Figure 2). As this is the first multi‐site palaeodietary study of early medieval populations from Wales and the Isle of Man, comparison between this study and data from other inland and coastal sites spanning the medieval period has the potential to elucidate meaningful similarities or differences that could relate to dietary trends or agricultural practices across time and location. As such, the palaeodietary results obtained as part of this study were compared with published data from other medieval populations from Britain including the 10th century cemetery of Ty Newydd, Bardsey Island (Arnold, 1998); the 5th–7th century Anglo‐Saxon cemetery of Berinsfield (Privat & O'Connell, 2001), the Iron Age/Norse site of Cnip, Orkney (Richards et al., 2001), 6th–11th century burials from Portmahomack (Curtis‐Summers et al., 2014), the 14th century Bordesley Abbey (Richards et al., 2001) and the 7th–17th century site of Auldhame, Scotland (Lamb et al., 2012). Figure 3 shows the mean published δ^13^C and δ^15^N values for the comparative datasets alongside the mean values obtained for the human remains analysed from each study site. The study sites have mean δ^13^C values that are lower than almost all the other comparative sites, while the mean δ^15^N values for the study sites are high in comparison to Berinsfield and Ty Newydd. The mean δ^15^N values for the study sites are more comparable to the values for Cnip and Bordesley Abbey, however, the mean δ^13^C values for the study sample are not as high as the mean δ^13^C values of these two comparative sites which are thought to reflect some marine‐resource consumption by those populations. It therefore seems that populations from western Britain, particularly those from the Isle of Man, consumed a terrestrial source of nitrogen‐enriched protein, but not marine resources. The combination of high nitrogen values and terrestrial carbon values is often seen as evidence for the consumption of omnivore protein (e.g., pig meat), freshwater resources (Müldner & Richards, 2005, p. 44), or the use of fertiliser since manure is known to enrich soil with ^15^N (Bogaard, Heaton, Poulton, & Merbach, 2007). In the context of this study, the use of fertiliser warrants further consideration particularly in light of the sulphur isotope data.

**Figure 4:**
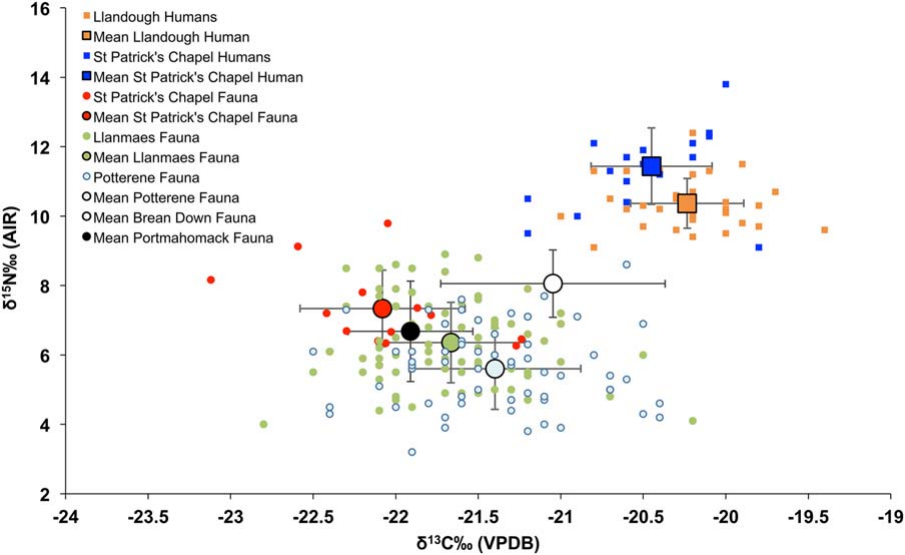
Comparison of δ^13^C_VPDB_ versus δ^15^N_AIR_ human and faunal data from St Patrick's Chapel (this study), Llandough (human) and Llanmaes (faunal) (Madgwick et al., 2012), Portmahomack (8th‐century faunal) (Curtis‐Summers et al., 2014), and Brean Down (faunal) (Britton, Müldner, & Bell, 2008)

There is a significant difference between the mean carbon isotope values of individuals from the east and west regions of our study area. Comparison of the faunal isotope data from St Patrick's Chapel and data from Madgwick, Mulville, and Stevens (2012) also reflects this regional difference (Figure 4). As the differences are evident in the human and faunal data, this suggests that the variability is due to environmental factors such as temperature, humidity, evaporation rate and rainfall, which all effect plant metabolism (Schmidt, Robins, & Werner, 2015).

**Figure 5:**
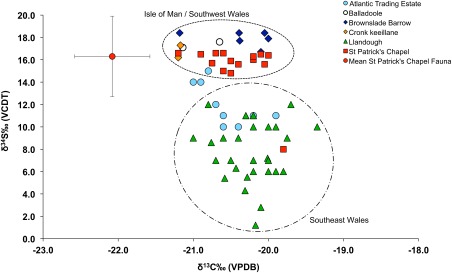
δ^13^C_VPDB_ versus δ^34^S_VCDT_ composition of the study sample illustrating the difference between the populations from southwest Wales/Isle of Man in comparison to the populations from southeast Wales

**Figure 6:**
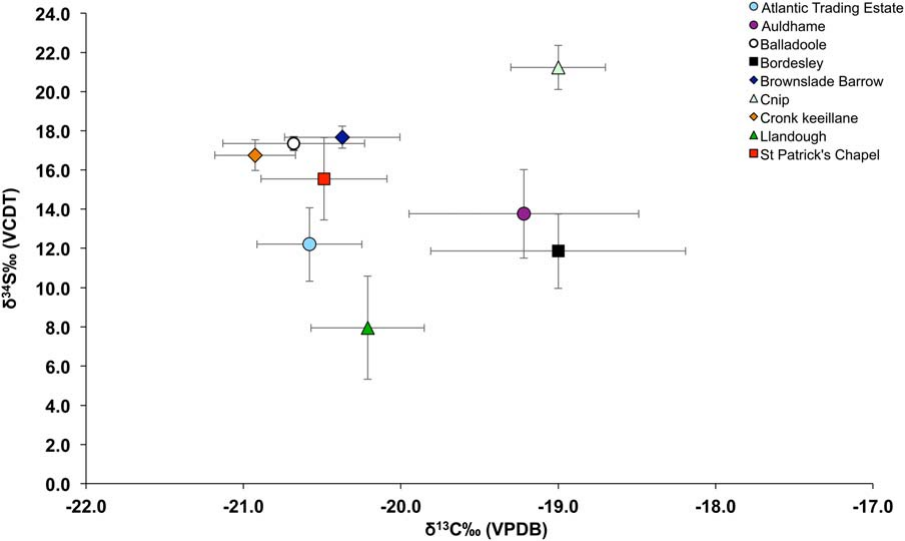
δ^13^C_VPDB_ versus δ^34^S_VCDT_ composition of the study sample compared with data published for human populations of medieval date from the British Isles (data from Lamb et al., 2012; Richards et al., 2001)

The analysis has revealed a distinct difference between the sulphur isotope values for those populations from the Isle of Man and southwest Wales in comparison to those populations from southeast Wales (Figure 5). The two populations from southeast Wales have sulphur isotope values within an “inland” population range (1–14‰) (Richards et al., 2006). By comparison, the populations from the Isle of Man and southwest Wales have high sulphur isotope values (>14‰), with some individuals—particularly from St Patrick's Chapel and Brownslade Barrow in Pembrokeshire—having δ^34^S values >17‰ and thus comparable to the range noted for marine organisms (17 to 21‰) (Lamb et al., 2012, p. 766; Richards et al., 2001, p. 186). As there is no evidence for the consumption of marine resources amongst these populations, and since seawater has a sulphur isotope value close to +20‰, the sulphur isotope values could reflect the influence of sea‐spray and the introduction of marine sulphates to the local biosphere of this region. The faunal δ^34^S data from St Patrick's Chapel is as high as the human δ^34^S data from St Patrick's Chapel and thus adds weight to the suggestion that exposure to sea‐spray is the cause of the high sulphur isotope values on the western coast and the Isle of Man. The sulphur isotope data from this study are compared to other published studies from the British Isles, and the values are in‐line with other coastal sites (Figure 6).

The use of seaweed as a fertilizer is also known to introduce marine sulphates to the soil resulting in high sulphur isotope values. Published strontium isotope data for individuals within the same study sample, including those from Pembrokeshire, reveals a number of individuals with high strontium concentrations (>100 ppm) (Hemer et al., 2013; Hemer et al., 2014), comparable to the strontium concentrations noted for populations from the Outer Hebrides where seaweed was used as a fertiliser (Montgomery & Evans, 2006; Montgomery, Evans, & Cooper, 2007, p. 1509). Indeed, other coastal farming communities from around the British Isles (e.g., Cornwall) and Ireland are known to have relied on seaweed for agricultural practices until the mid‐20th century (Amorosi et al., 1998; Balasse, Tresset, Dobney, & Ambrose, 2005; Hallsson, 1961). It is therefore necessary to consider the use of seaweed as a fertiliser by the populations from western Britain to explain the occurrence of high sulphur isotope values. There is also further evidence for this from the archaeological record. Analysis of soil samples from Brownslade Barrow revealed the presence of clover‐type weeds, and it was suggested that the sandy soils around Brownslade Barrow were of low nutrient content, and therefore it was necessary to fertilize the land with household waste or seaweed ash (Carruthers, 2012). The use of seaweed ash as a fertilizer is further suggested by the recovery of charred seaweed remains belonging to the *Fucus* species from the site (Carruthers, 2012). It is therefore possible that populations in and around the southwest coast of Wales, and possibly the Isle of Man, utilised seaweed as a fertiliser for crops and may explain the occurrence of elevated sulphur isotope values observed amongst the populations from this region by comparison to those “inland” populations from the southeast. In addition, seaweed may have been consumed directly by the coastal inhabitants.

That a distinction can be observed between the sulphur isotope values of those populations from the southwest coast of Wales and the Isle of Man in comparison to the two populations from southeast Wales may also provide a means by which to distinguish individuals of non‐local origin. For example, one individual (SP 216) buried at St Patrick's Chapel has a sulphur isotope value (δ^34^S= 8‰) more consistent with the values recorded for Llandough and Atlantic Trading Estate. This individual also stands out in terms of their strontium isotope value (0.712), and suggests that this adult female moved from east Wales to the region around St Patrick's Chapel on the Pembrokeshire coast. Similarly, four females buried at Atlantic Trading Estate have sulphur isotope values close to the values observed for the populations from southwest Wales, and raises the possibility that these women moved from a coastal region in Pembrokeshire to Atlantic Trading Estate in the southeast.

The difference between the sulphur isotope values of the populations from the west coast of Wales and the Isle of Man in comparison to those from southeast Wales stems from the fact that Llandough has the lowest mean sulphur isotope value, and the lowest recorded sulphur isotope values for the entire study sample (Tables 1 and 4). Of all the sites within the study sample, Llandough is the furthest away from the sea, and the region may have been under less influence from atmospheric marine sulphates, while seaweed may not have been used as a fertiliser or consumed by those living in this region. Such regional differences in the sulphur isotope values evident here lends support to Richards et al.'s (2001) suggestion that sulphur isotopes may offer a palaeomobility signal.

While geographical location and proximity to the sea and its resources is likely to be the primary factor influencing the regional differences in sulphur isotopes, individuals buried at Llandough may also have been consuming produce that was grown further inland and was available to those communities living at, or nearby to, this ecclesiastical centre. Historical evidence demonstrates that religious foundations in southeast Wales were surrounded by land used to support members of the lay and religious communities; for example, the monastic community of Llancarfan grazed their sheep on the island of Flatholm (Davies, 1982, p. 164), while a charter entry from AD 620 notes that the monastery near Caerwent was supported by its *ager suburbanus*, that is, the fields outside the city (Davies, 1982, p. 164). While some religious communities were self‐supporting, other ecclesiastical centres owned land that was farmed and managed by members of the lay community who took some of the produce to meet their own needs, and provided the remainder to the monastery (Davies, 1982, p. 165). Some of the more powerful religious houses, like the bishopric of Llandaff, acted as landlords and were capable of extorting surplus produce in the form of food renders—including livestock, bread and ale—from very distant churches and their communities (Davies, 1982, p. 165). There is also the connection between religious houses and nearby high‐status settlements, and the relationship that this created in terms of the collection and redistribution of surplus produce. For instance, Llandough is connected to the high‐status settlement of Dinas Powys, less than 3 km away (Holbrook & Thomas, 2005). Dinas Powys has yielded a considerable volume of archaeozoological material as well as evidence for specialist craft working and imported Mediterranean pottery (Alcock, 1963; Seaman, 2013). Such evidence led to the suggestion that Dinas Powys was an elite stronghold that received tribute from local tenants in the form of food renders, including mature, fattened animals (Alcock, 1963; Gilchrist, 1988, p. 59). As such, the elite at Dinas Powys enjoyed a position within society which allowed them to accrue local agricultural surplus which in turn was consumed, alongside imported Mediterranean foodstuffs, as part of frequent, large‐scale feasting events which were central to the establishment and maintenance of allegiances between elites and their subjects (Davies, 1982; Seaman, 2013). The presence of the same Mediterranean amphorae at Llandough demonstrates that secular elites residing at Dinas Powys also made donations of imported consumables—such as wine and oil—to their neighboring religious community, and would benefit from the obliged counter‐gift from the monastic brethren (e.g., prayers for the soul of the patron and his family) (Grierson, 1959, p. 137; Knight, 2005, p. 104). The donation of luxury items to the monastic community at Llandough raises the possibility that other agricultural produce collected as food renders by the elite of Dinas Powys may have also been passed on to its associated religious community at Llandough. The low sulphur isotope values recorded for the sample from this cemetery may therefore reflect individuals who consumed foodstuffs drawn from the hinterland.

Since Llandough was an important religious foundation in southeast Wales during the early medieval period, it is also necessary to consider the possibility that the cemetery served communities who did not live in the immediate vicinity of the coast. Indeed, a previous investigation of population mobility in Wales identified individuals buried at Llandough who may have grown up in other parts of Wales and the borders with England suggesting some degree of movement to the Llandough region (Hemer et al., 2013, p. 2356). As such, the low mean sulphur isotope value for individuals buried at Llandough may reflect the fact that this monastery was well‐connected to populations from further inland, and that the cemetery was a foci of burial for a far‐reaching Christian community. By contrast, the cemeteries from southwest Wales and the Isle of Man served only local, coastal communities who relied on the consumable resources they were able to grow in their immediate vicinity.

## 5 Conclusions

This study aimed to shed light on the use and consumption of dietary resources by communities living in Wales and the Isle of Man during the early medieval period. A limited amount of settlement and faunal evidence meant that the opportunity to investigate diet through stable isotope analysis would provide an invaluable perspective on the communities from western Britain. As such, the first multi‐site investigation was undertaken, using carbon, nitrogen, and sulphur isotope analysis. Despite many of the sites being in close proximity to the coast, the results revealed an overall reliance on terrestrial protein. Clear differences were observed, however, between the populations from the west coast of Wales and the Isle of Man in comparison to the populations from southeast Wales. A clear distinction was identified in the sulphur isotope values between these populations, with those from the west having a distinctly “marine” sulphur signature, while those from the east had a more “inland” signature. It was proposed that this difference may relate to different subsistence strategies employed by populations from these regions, and consideration is given to the possibility that those in the west were reliant on the use of seaweed. Moreover, consideration was given to the fact that the cemetery sites themselves served very different communities; Llandough was an important ecclesiastical centre which was well‐connected to communities in and around the southeast including the elites occupying the high‐status settlement of Dinas Powys. As such, there was access to resources, and people, from a much‐wider geographical area. In contrast, the cemeteries from the west coast were the foci for local communities who did not have the same networks as the large ecclesiastical centres, and as such these local populations relied far more on produce grown in and around the vicinity of the coast.
